# Fine-tuning of synaptic upscaling at excitatory synapses by endocannabinoid signaling is mediated via the CB1 receptor

**DOI:** 10.1038/srep16257

**Published:** 2015-11-06

**Authors:** Yunping Song, Jian Zhang, Chu Chen

**Affiliations:** 1 Neuroscience Center of Excellence, School of Medicine, Louisiana State University Health Sciences Center, New Orleans, LA 70112, USA

## Abstract

The endocannabinoid 2-arachidonoylglycerol (2-AG) functions as a retrograde signaling molecule mediating synaptic transmission and plasticity at both inhibitory and excitatory synapses. However, little is known about whether 2-AG signaling is involved in homeostatic regulation of miniature synaptic events at excitatory synapses in response to activity deprivation. Here, we report that chronic blockade of firing by tetrodotoxin (TTX) for two days resulted in increases both in the frequency and amplitude of spontaneous miniature excitatory postsynaptic currents (mEPSCs) in cultured mouse hippocampal neurons. However, treatment with 2-AG alone or JZL184, a potent and selective inhibitor for monoacylglycerol lipase (MAGL) that hydrolyzes 2-AG, induced a CB1 receptor-dependent reduction of the frequency of mEPSCs, but not the amplitude. The TTX-increased frequency was blunted by 2-AG or JZL184 and this effect was eliminated by pharmacological or genetic inhibition of CB1 receptors. In addition, TTX still increased frequency and amplitude of mEPSCs in the presence of CB1 receptor inhibition. Our results suggest that while endocannabinoids are not required for induction of synaptic scaling at excitatory glutamate synapses after chronic activity deprivation, 2-AG signaling may play a role in fine-tuning of synaptic strengths via presynaptically-expressed CB1 receptors.

Synaptic scaling is a form of homeostatic synaptic plasticity that plays an important role in stabilizing the excitability of neural circuits in response to the deprivation of excitatory or inhibitory neurotransmission (Turrigiano, 2008)^1^,^2^. Turrigiano *et al.*^3^ first described activity dependent scaling of synaptic strength in cultured neocortical neurons, where chronic blockade of activity by tetrodotoxin (TTX), a selective voltage-gated sodium blocker, or CNQX, an AMPA receptor blocker, increases the amplitude of miniature excitatory postsynaptic currents (mEPSCs). Increase in mEPSCs by chronic suppression of neural excitability in cultures appear to result from an up-regulation of postsynaptic glutamate receptors^4^,^5^,^6^, and/or an increase of presynaptic glutamate release^5^,^6^. It has been reported that the strengthening of miniature excitatory postsynaptic currents (mEPSCs) after exposure to TTX for two days in a rat hippocampal culture system was associated with increases in the length of dendrites and number of synapses^7^. The accumulation of GluA1-containing AMPARs at postsynaptic terminals were also observed in cultured hippocampal neurons as a consequence of homeostasis regulation^8^,^9^. In addition, inhibitory synapses are also affected under activity deprivation. For instance, the amplitude or frequency of miniature inhibitory postsynaptic currents (mIPSCs) is reduced in tonic presence of TTX^10^,^11^,^12^. The absence of excitatory neurotransmission affects both inhibitory and excitatory synapses and the changes in synaptic activity occur at presynaptic and/or postsynaptic sites^3^. Thus, synaptic scaling represents an important homeostatic regulation of synaptic activity.

While homeostatic regulation of synaptic plasticity has been extensively studied, the signaling mechanisms that regulate or tune synaptic scaling are not fully understood^1^,^2^. Endocannabinoids (eCBs) are naturally occurring lipid mediators regulating synaptic transmission and plasticity at both excitatory and inhibitory synapses^13^. Regulation of synaptic activity by eCBs is through retrograde signaling: eCBs are released from postsynaptic cells and act on presynaptically-expressed CB1 receptors (CB1R), resulting in a reduction of the release probability of neurotransmitters, including GABA and glutamate^13^. The eCB system has been proposed to play important roles in homeostatic regulation of brain functions in physiology and disease^14^,^15^,^16^. Kim & Alger^17^ demonstrated previously that homeostatic down regulation of synaptic scaling at specific inhibitory synapses caused by chronic blockade of neuronal firing is associated with facilitation of anandamide uptake and degradation. The study of Zhang *et al.*^9^, showed that suppression of mIPSCs in response to the acute absence of mEPSCs (1–3 hours) is dependent on CB1R-mediated 2-arachidonoylglycerol (2-AG) signaling. This suggests that eCBs are involved in scaling of inhibitory synaptic strengths when neural firing or excitatory synaptic activity is absent. However, it is still not clear whether 2-AG signaling plays a role in synaptic upscaling at excitatory synapses upon chronic activity deprivation. Here we show that endocannabinoids are not required for induction of synaptic scaling in cultured hippocampal neurons treated with TTX for two days. However, the TTX-increased frequency, but not the amplitude of mEPSCs, was diminished when 2-AG signaling was enhanced. The effect of synaptic scaling down by 2-AG was eliminated by pharmacological or genetic inhibition of CB1R. Our results suggest that 2-AG signaling may play a role in CB1R-mediated fine-tuning of synaptic strengths at excitatory synapses when neural network activity is chronically deprived.

## Results

### Increase in frequency of mEPSC by chronic treatment of TTX is blunted by 2-AG signaling

**Figure 1:**
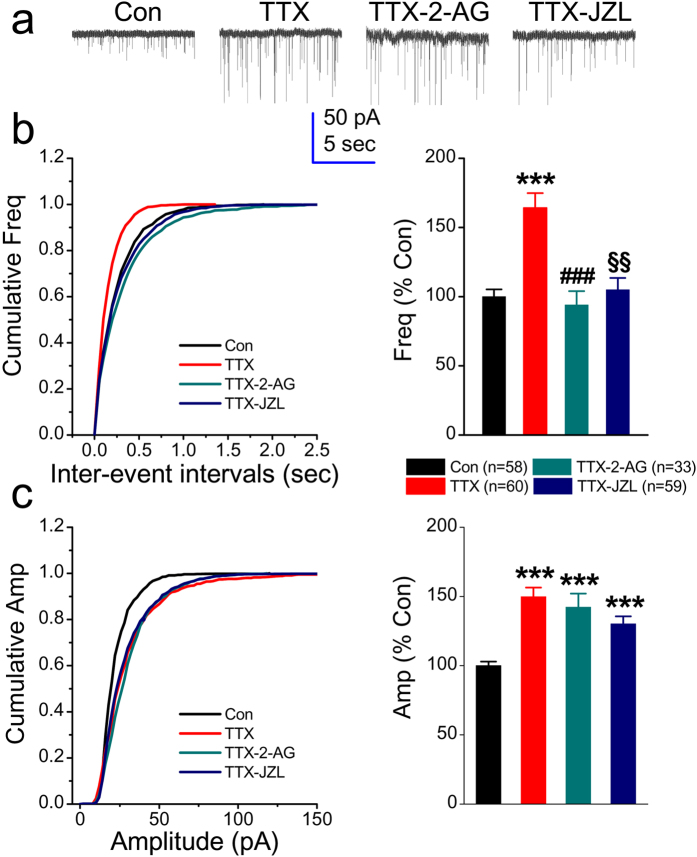
Increase in the frequency of mEPSCs by chronic treatment with TTX in cultured hippocampal neurons is blunted by the exogenous and endogenous endocannabinoid 2-arachidonylglycerol (2-AG). (**a**) Representative recording traces of mEPSCs under various treatments. The whole-cell patch clamp recordings were made in cultured mouse hippocampal neurons that were treated with TTX (1 μM) and JZL184 (2 μM) for two days or 2-AG (3 μM) once a day for two days. Endogenous metabolism of 2-AG was inhibited by JZL184, a highly selective and potent inhibitor of monoacylglycerol lipase (MAGL) that hydrolyzes 2-AG. All the values of recordings from the cultures with different treatments are normalized to that from the control recorded in the same day. (**b**) Cumulative probability of mEPSC frequency recorded in neurons with different treatments (left) and mean percentage of changes in the frequency of mEPSCs in neurons with different treatments (right). (**c**) Cumulative probability of mEPSC amplitude recorded in neurons with different treatments (left) and mean percentage of changes in the amplitude of mEPSCs in neurons with different treatments (right). Data are mean ± SEM. The number in the parentheses represents number of neurons recorded from 2 to 6 different batches of cultures. ***p < 0.001, compared with the control; ^###^*p* < 0.01, ^§§^*p* < 0.01, compared with TTX (the Dunn’s post hoc test following the Kruskal-Wallis test).

To investigate whether 2-AG signaling is required in homeostatic regulation of synaptic strength in response to persistent absence of neuronal activity, whole-cell patch clamp recordings were made to detect mEPSCs in cultured mouse hippocampal neurons (from days *in vitro* 10 to 15). As shown in Fig. 1, both the frequency and amplitude of mEPSC were significantly increased in culture treated with TTX (1 μM) for two days (164 ± 11%, *p* < 0.001 and 149 ± 7%, *p* < 0.001, respectively). This is in agreement with the results reported by others^18^,^19^.

To determine whether 2-AG regulates scaling of synaptic strength induced by activity deprivation, 2-AG (3 μM) was added to the cultures once a day for two days in the presence of TTX. While TTX still increased the amplitude of mEPSCs in cultures treated with 2-AG (142 ± 9%, *p* < 0.001), 2-AG occluded the TTX-induced increase in the frequency (164 ± 11% versus 94 ± 10%, *p* = 0.001). This indicates that the action of 2-AG on synaptic activity is via a presynaptic mechanism. 2-AG is primarily hydrolyzed by the enzyme monoacylglycerol lipase (MAGL). To confirm the reversal of TTX-increased frequency of mEPSCs by 2-AG, we treated cultures with JZL184 (2 μM), a high selective and potent inhibitor for MAGL^20^, to raise the levels of endogenously-released 2-AG. As expected, JZL184 reversed the TTX-elevated frequency of mEPSCs to the control level, but not the amplitude (Frequency: 105 ± 8% and amplitude: 130 ± 5%). These results suggest that scaling of synaptic strength after chronic activity deprivation can be fine-tuned by 2-AG signaling at excitatory glutamate synapses.

### Suppression of synaptic activity by 2-AG is mediated through CB1R

**Figure 2:**
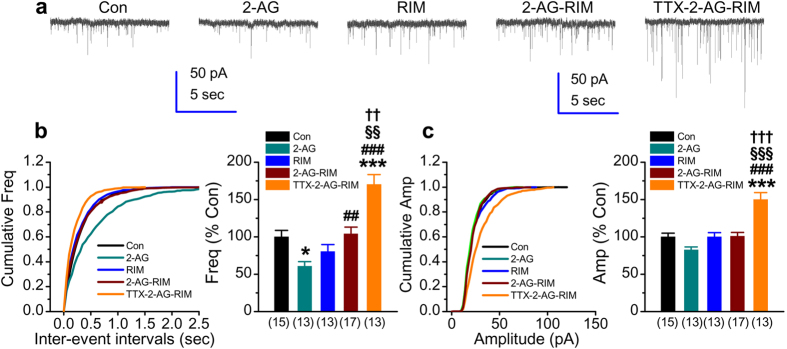
CB1 receptor mediates 2-AG-induced suppression of the frequency of mEPSCs. (**a**) Representative recording traces of mEPSCs under various treatments. Rimonabant (RIM, 1 μM) was added to cultures for two days. The treatment with TTX and 2-AG was the same as that described in Fig. 1. (**b**) Cumulative probability of mEPSC frequency recorded in neurons with different treatments (left) and mean percentage of changes in the frequency of mEPSCs in neurons with different treatments (right). (**c**) Cumulative probability of mEPSC amplitude recorded in neurons with different treatments (left) and mean percentage of changes in the amplitude of mEPSCs in neurons with different treatments (right). Data are mean ± SEM. *p < 0.05, ***p < 0.001, compared with the control; ^##^*p* < 0.01, ^###^*p* < 0.001, compared with 2-AG; ^§§^*p* < 0.01, ^§§§^*p* < 0.001, compared with RIM; ^††^*p* < 0.01, ^†††^*p* < 0.001, compared with 2-AG + RIM (ANOVA with Dunn’s post hoc test).

It has been reported previously that acute application of 2-AG reduces the frequency of mEPSCs in cultured hippocampal neurons^21^. However, it is still not clear about whether mEPSCs is also affected by chronic treatment with 2-AG. To this end, mEPSCs were recorded in cultures treated 2-AG once a day for two days. As shown in Fig. 2, chronic treatment of 2-AG reduced the frequency, but not the amplitude of excitatory spontaneous events (frequency: 61 ± 6%, *p* = 0.01, amplitude: 83 ± 4%, *p* = 0.16).

**Figure 3:**
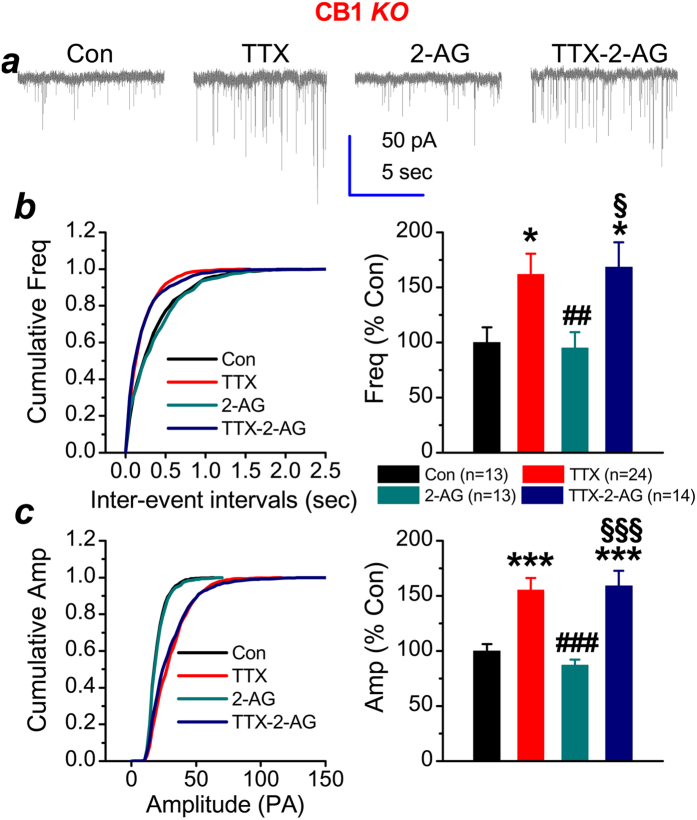
Chronic activity deprivation increases miniature spontaneous events and 2-AG fails to suppress them in cultured neurons lacking CB1R. (**a**) Representative recording traces of mEPSCs under various treatments. (**b**) Cumulative probability of mEPSC frequency recorded in neurons with different treatments (left) and mean percentage of changes in the frequency of mEPSCs in neurons with different treatments (right). (**c**) Cumulative probability of mEPSC amplitude recorded in neurons with different treatments (left) and mean percentage of changes in the amplitude of mEPSCs in neurons with different treatments (right). Data are mean ± SEM. *p < 0.05, ***p < 0.001, compared with the control; ^##^*p* < 0.01, ^###^*p* < 0.001, compared with TTX; ^§^*p* < 0.05, ^§§§^*p* < 0.001, compared with TTX-2-AG (ANOVA with Dunn’s post hoc test).

Modulation of synaptic activity by eCB signaling is primarily mediated through presynaptically-expressed CB1R^13^. To determine whether decrease in the frequency of mEPSCs by 2-AG is mediated via CB1R, Rimonabant (RIM), a selective CB1R antagonist, was used to block the activity of CB1R. While RIM (1 μM) alone did not significantly alter both the frequency and amplitude of mEPSCs, it blocked the 2-AG-reduced frequency (Fig. 2). In addition, CB1R inhibition by RIM also eliminated the suppressive effect of 2-AG on TTX-elevated frequency of mEPSCs, but not the amplitude (frequency: 170 ± 13%, *p* = 0.001, amplitude: 150 ± 9%, *p* < 0.001). These results from pharmacological inhibition of CB1R indicate that 2-AG-reduced the frequency of mEPSCs is likely mediated via CB1R. To further confirm this speculation, we cultured hippocampal neurons from CB1R knockout (KO) mice. As shown in Fig. 3, chronic activity deprivation by TTX still increased both the frequency and amplitude of mEPSCs in cultured neurons deficiency in CB1R (frequency: 162 ± 19%, *p* = 0.02, amplitude: 155 ± 11%, *p* < 0.001), suggesting that induction of synaptic scaling does not required endocannabinoid signaling. Similar to the effect of RIM, 2-AG failed to reduce the frequency of mEPSCs in neurons lacking CB1R (Fig. 2). Likewise, the suppressive effect of 2-AG on TTX-elevated activity was prevented in cultured neurons lacking CB1R (frequency: 168 ± 22%, *p* = 0.009, amplitude: 160 ± 14%, *p* < 0.001). This further confirms that reduction of frequency of mEPSCs by 2-AG is mediated via presynaptically-expressed CB1R.

**Figure 4:**
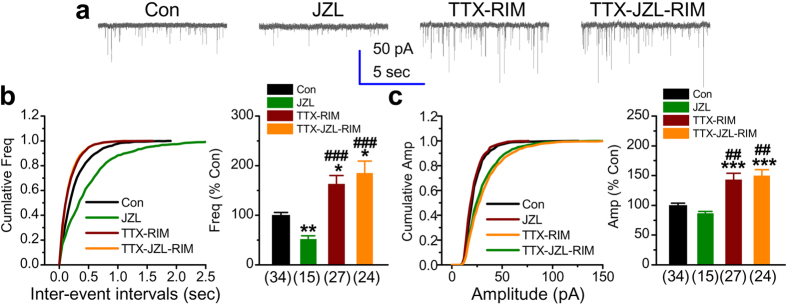
Pharmacological inhibition of CB1R does not alter scaling up of synaptic activity by chronic TTX treatment, but blocks the suppressive effect of endogenous 2-AG on TTX- increased frequency of mEPSCs. (**a**) Representative recording traces of mEPSCs under various treatments. The level of endogenous 2-AG was raised by inhibition of MAGL with JZL184 (2 μM). (**b**) Cumulative probability of mEPSC frequency recorded in neurons with different treatments (left) and mean percentage of changes in the frequency of mEPSCs in neurons with different treatments (right). (**c**) Cumulative probability of mEPSC amplitude recorded in neurons with different treatments (left) and mean percentage of changes in the amplitude of mEPSCs in neurons with different treatments (right). Data are mean ± SEM. *p < 0.05, **p < 0.01, ***p < 0.001, compared with the control; ^##^*p* < 0.01, ^###^*p* < 0.001, compared with JZL184 (the Dunn’s post hoc test following the Kruskal-Wallis test).

To explore whether endogenously-released 2-AG exhibits a similar effect on miniature spontaneous activity of excitatory glutamate receptors to that of exogenously-applied 2-AG, JZL184 was again used to raise endogenous 2-AG levels in culture. As seen in Fig. 4, treatment with JZL184 (2 μM) for 2 days resulted in a significant decrease in the frequency of mEPSC (51 ± 7%, *p* = 0.004), similar to the effect of 2-AG. Also, the amplitude was not affected by JZL184 (86 ± 4%, *p* = 0.77). Interestingly, chronic treatment with TTX still increased both the frequency and amplitude of mEPSCs in the presence of CB1R blockade (frequency: 163 ± 17%, *p* = 0.04, amplitude: 143 ± 11%, *p* = 0.008). This is consistent with the results shown in Fig. 3, where TTX elevated the frequency and amplitude of mEPSCs in neurons lacking CB1R. This supports the fact that endocannabinoids are not required for the induction of synaptic scaling by chronic activity deprivation. In addition, as shown in Fig. 4, suppression of TTX-increased frequency of mEPSCs by inhibition of 2-AG metabolism was diminished by inhibition of CB1R (frequency: 185 ± 25%, *p* = 0.03, amplitude: 149 ± 10%, *p* < 0.001). These results indicate that endogenously-released 2-AG is able to tune synaptic strengths in the absence of presynaptic activity.

**Figure 5:**
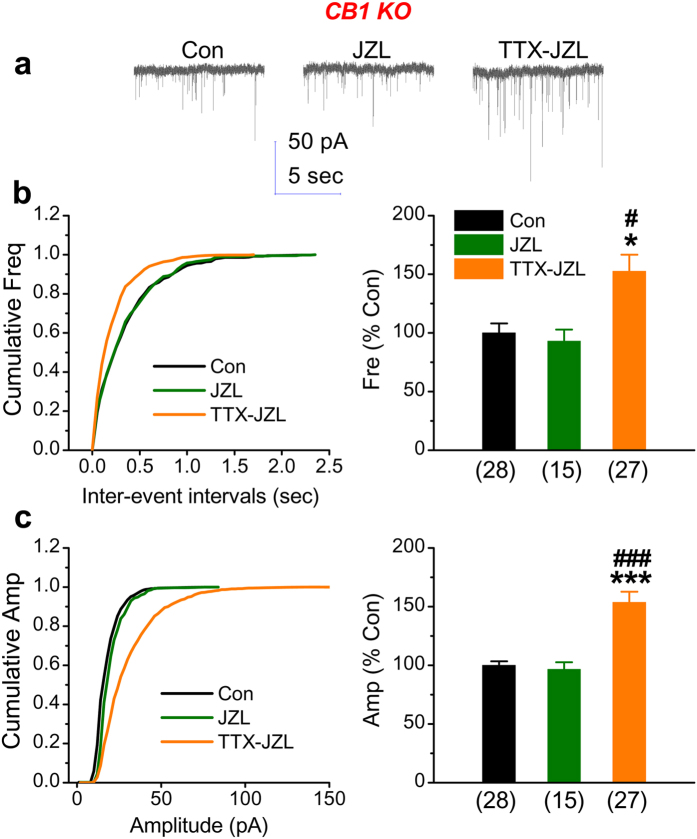
Genetic inhibition of CB1R prevents the suppressive effect of endogenous 2-AG on TTX-increased activity of mEPSCs. (**a**) Representative traces of mEPSCs recorded in cultured mouse hippocampal neurons lacking CB1R under various treatments. Endogenous synthesized 2-AG was preserved by JZL184 (2 μM). (**b**) Cumulative probability of mEPSC frequency recorded in neurons with different treatments (left) and mean percentage of changes in the frequency of mEPSCs in neurons with different treatments (right). (**c**) Cumulative probability of mEPSC amplitude recorded in neurons with different treatments (left) and mean percentage of changes in the amplitude of mEPSCs in neurons with different treatments (right). Data are mean ± SEM. *p < 0.05, ***p < 0.001, compared with the control; ^#^*p* < 0.05, ^###^*p* < 0.001, compared with JZL184 (ANOVA with Dunn’s post hoc test).

To further verify that the effect of endogenous 2-AG on spontaneous excitatory events is mediated via CB1R, hippocampal neurons deficiency in CB1R were treated with JZL184 (2 μM). As shown in Fig. 5, inhibition of MAGL by JZL184 did not affect both the frequency and amplitude of mEPSCs. Moreover, JZL184 also failed to suppress the increase in the frequency of mEPSCs by TTX (frequency: 153 ± 14%, *p* = 0.02). These results confirm that the effect of endogenously-released 2-AG on the frequency of mEPSCs is mediated through a CB1R-dependent mechanism.

### Anandamide induces a similar effect to that of 2-AG on tuning synaptic scaling at excitatory synapses

**Figure 6:**
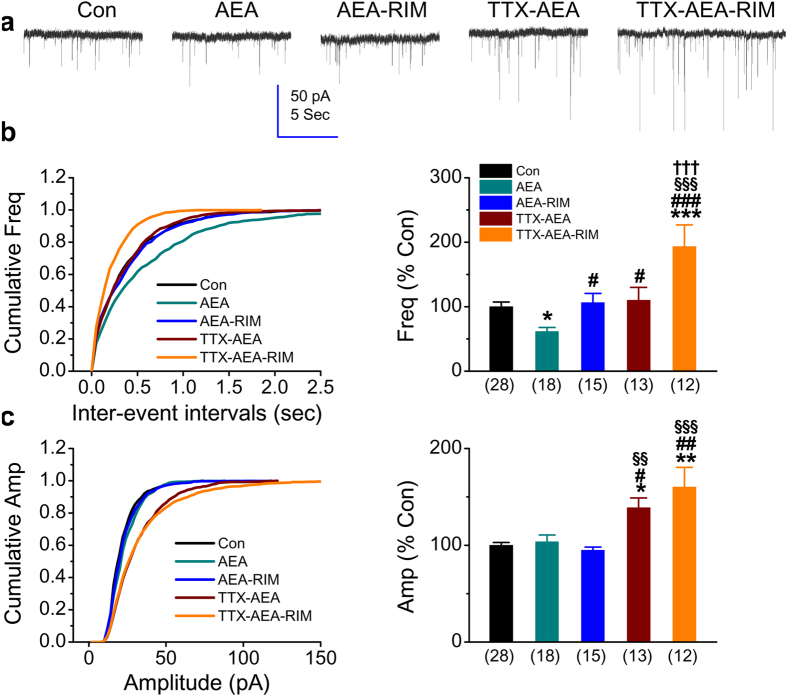
CB1 receptor mediates AEA-induced suppression of the frequency of mEPSCs. (**a**) Representative recording traces of mEPSCs under various treatments. TTX (1 μM) and Rimonabant (RIM, 1 μM) was added to cultures for two days. Anandamide (AEA, 5 μM) was treated in culture once a day for two days. (**b**) Cumulative probability of mEPSC frequency recorded in neurons with different treatments (left) and mean percentage of changes in the frequency of mEPSCs in neurons with different treatments (right). (**c**) Cumulative probability of mEPSC amplitude recorded in neurons with different treatments (left) and mean percentage of changes in the amplitude of mEPSCs in neurons with different treatments (right). Data are mean ± SEM. *p < 0.05, **p < 0.01, ***p < 0.001, compared with the control; ^#^*p* < 0.05, ^##^*p* < 0.01, ^###^*p* < 0.001, compared with AEA; ^§§^*p* < 0.01, ^§§§^*p* < 0.001, compared with AEA + RIM; ^†††^*p* < 0.001, compared with TTX + AEA (ANOVA with Dunn’s post hoc test).

Anandamide (AEA) is the first-identified endocannabinoid. It has been shown previously that AEA plays an important role in homeostatic downscaling for inhibitory synapses^17^. To test whether AEA affects synaptic scaling at excitatory synapses, we treated cultures with AEA in the absence and presence of TTX or RIM. As seen in Fig. 6, treatment of AEA (5 μM) alone once a day for 2 days significantly reduced the frequency of mEPSC (62 ± 6% of control, *p* < 0.05), but not the amplitude (104 ± 7%, *p* = 0.99). The decrease in frequency of mEPSC by AEA was blocked by RIM (*p* < 0.05). Similar to the effect of 2-AG, AEA also reversed the increase in the frequency of mEPSCs by chronic TTX treatment, but not the amplitude. This effect was also mediated via CB1R since the frequency of mEPSCs in recordings from the TTX-AEA-RIM group was significantly elevated compared to that of the control (193 ± 33%, *p* < 0.001).

## Discussion

In this report, we show that endocannabinoid signaling is not required for the induction of synaptic scaling by providing the evidence that both the frequency and amplitude of mEPSCs in cultured mouse hippocampal neurons were elevated by chronic deprivation of firing activity in the absence and presence of CB1R inhibition. However, exogenous and endogenous 2-AG reduced the frequency of mEPSCs under both normal and activity deprived conditions through a CB1R-dependent mechanism. We also observed that AEA exerts the similar effects at that of 2-AG. This suggests that endocannabinoid signaling may play an important role in fine-tuning of synaptic strengths when synaptic activity is scaled up in response to persistent absence of neural firing.

Synaptic scaling is an important stabilizing mechanism that may represent a general form of activity-dependent regulation of synaptic transmission. We observed in the present study that miniature spontaneous activity at glutamatergic synapses was scaled up by chronic treatment of TTX for two days. This is consistent with the reports by others^3^,^6^,^18^,^19^. Changes in synaptic strengths in response to the blockade of neuronal activity may be associated with increases in the density of excitatory synaptic contacts^18^, glutamate release probability^22^,^23^,^24^ or postsynaptic AMPA receptor expression and trafficking^1^,^5^ (Turrigiano, 2008). However, it is not clear about which signaling molecules that regulate synaptic strength (*e.g.*, scaling up or down) when neuronal activity is altered. A few molecules have been proposed to play a role in synaptic scaling^1^. For instance, it has been demonstrated that glial tumor necrosis factor-α (TNFα) is critical for scaling up of synapses during prolonged activity blockade by maintaining synapses in a plastic state^25^,^26^. eCBs function as a retrograde messenger regulating synaptic transmission and plasticity both at inhibitory and excitatory synapses through a presynaptically-expressed CB1R^13^ and likely play a role in synaptic scaling. It has been shown that eCB signaling is involved in homeostatic down regulation of synaptic scaling of GABAergic synapses by chronic blockade of neuronal firing^17^. Zhang *et al.*^9^ reported that 2-AG signaling is essential for a down-regulation of the mIPSC frequency in response to the acute (1 to 3 hours) absence of mEPSCs^13^. However, it is not well established as to whether eCBs contribute to synaptic upscaling at excitatory synapses following chronic activity deprivation. In the present study, we observed that the frequency and amplitude of mEPSCs were increased by chronic deprivation of neuronal activity in cultures where CB1R was pharmacologically or genetically inhibited, implying that eCB signaling is not required for homeostatic regulation of synaptic strengths at excitatory synapses upon chronic TTX treatment. This suggests that homeostatic regulation of synaptic strengths at excitatory synapses by eCB signaling may be different from that at inhibitory synapses^9^,^17^. For instance, CB1R is predominantly expressed on nerve terminals of inhibitory synapses. Thus, modulation of synaptic activity by endocannabinoid signaling is greater at GABAergic synapses than that at glutamatergic synapses. This may be associated with significant effects of endocannabinoid signaling in regulation of synaptic scaling as reported previously^9^,^17^. However, we did observe that the increase in the frequency of mEPSCs by TTX was blunted by both exogenously-applied and endogenously-released 2-AG at excitatory synapses and that this effect was eliminated by inhibition of CB1R. This effect was mimicked by AEA. This suggests that endocannabinoid signaling may play a role in fine-tuning of synaptic strength at glutamate synapses after prolonged deprivation of neuronal activity through a CB1R-dependent mechanism. While our observations of synaptic scaling after neuronal firing deprivation were made in cultured mouse hippocampal neurons, an *in vitro* model that is similar to that in cortical neuronal culture initially observed by^1^, further studies in brain slices would gather more important information to illustrate endocannabinoid signaling in local fine-tuning of synaptic scaling of excitatory synapses. Although endocannabinoid signaling is important in maintaining brain homeostasis via different pathways in physiology and disease, our results provide evidence that endocannabinoid signaling is not required for the induction of synaptic upscaling of excitatory glutamatergic synapses in responses to changes in neuronal activity.

## Methods

### Primary hippocampal neuron culture

Primary hippocampal neurons were cultured as described previously^21^,^27^,^28^,^29^,^30^. Briefly, mouse (normal and CB1R knockouts) pups at postnatal day 0 were rapidly decapitated, and hippocampi were immediately dissected out from the brain. The tissue was incubated in oxygenated trypsin for 10 min at 37 °C and then mechanically triturated. Cells were spun down and resuspended in Neurobasal–B27 medium (Invitrogen) supplemented with 0.5 mml-glutamine, penicillin–streptomycin and 25 μm glutamate. Cells (1 × 10^6^) were loaded into poly d-lysine-coated 35 mm culture dishes. One-third to one-half of the culture medium without glutamate was changed every 3 days. Cultures were used between 10 and 15 days *in vitro* (DIV). All the experiments were performed in compliance with the US Department of Health and Human Services Guide for the Care and Use of Laboratory Animals. The care and use of the animals reported and experimental protocols in this study were approved by the Institutional Animal Care and Use Committee of Louisiana State University Health Sciences Center.

### Electrophysiological recordings

Miniature excitatory postsynaptic currents (mEPSCs) were recorded in hippocampal neurons in culture under voltage clamp using an Axopatch-200B amplifier as describe previously^21^,^28^,^29^. Recording pipettes (3–5 MΩ) were pulled from borosilicate glass with a micropipette puller (Sutter Instrument Co.). The internal pipette solution contained (mm): 115.0 caesium gluconate, 15.0 CsCl, 4.0 NaCl, 10.0 Hepes, 0.5 EGTA, 4.0 Mg_2_ATP, and 0.5 Na_2_GTP (pH 7.25 with CsOH). The membrane potential was held at −70 mV. The external solution contained (mm): 130.0 NaCl, 2.5 KCl, 1.0 MgCl_2_, 10.0 Hepes, 1.25 NaH_2_PO_4_, 2.0 CaCl_2_, 25.0 glucose (pH 7.4 with NaOH). To isolate mEPSCs, TTX (0.5 μM), a voltage-gated Na^+^ channel blocker, bicuculline (10 μm), an competitive antagonist of GABA_A_ receptors, were included in the external solution. All experiments were performed at room temperature (22–24 °C). The frequency, amplitude and kinetics were analyzed using the MiniAnalysis program.

### Chemicals

2-AG, AEA, and AM251 were purchased from Cayman Chemical (Ann Arbor, MI, USA). JZL184 and Rimonabant (SR141716) were provided by National Institutes of Health Mental Health Institute Chemical Synthesis and Drug Supply Program. TTX was purchased from Sigma-Aldrich (St. Louis, MO, USA). All other drugs and chemicals were obtained from Sigma-Aldrich unless stated otherwise. Reagents were dissolved in DMSO to make stock solutions at concentrations of 50–100 mM and distributed in small vials. To prevent the oxidation of lipids, the air in vials was expelled with nitrogen gas before being stored in a −80 °C freezer. The stock solutions in the vials were diluted with the external solution to desired concentrations just before recordings. To rule out potentially non-specific effects of the solvents, the same amount of DMSO was included in the control external solution.

### Data analysis

Data are presented as means ± SEM unless stated otherwise. Spontaneous synaptic activity in culture changes with days *in vitro*. To correct this variation, the values of all the recordings made in each day from different treatments were normalized to that from the control recorded on the same day. The Kolmogorov-Smirnov test was used to explore the normality of each dataset. ANOVA with *post hoc* tests were used for parametric comparison, while Dunn’s post hoc test following the Kruskal-Wallis test were used for statistical comparison. Differences were considered significant when *p* < 0.05.

## Additional Information

**How to cite this article**: Song, Y. *et al.* Fine-tuning of synaptic upscaling at excitatory synapses by endocannabinoid signaling is mediated via the CB1 receptor. *Sci. Rep.* **5**, 16257; doi: 10.1038/srep16257 (2015).
